# The complete chloroplast genome sequence of *Convallaria majalis* L.

**DOI:** 10.1080/23802359.2022.2067501

**Published:** 2022-04-22

**Authors:** Wen-Xiao Men, Yue-Yue Song, Yan-Ping Xing, Che Bian, He-Fei Xue, Liang Xu, Ming Xie, Ting-Guo Kang

**Affiliations:** School of Pharmacy, Liaoning University of Traditional Chinese Medicine, Dalian, China

**Keywords:** Asparagaceae, chloroplast genome, *Convallaria majalis*, phylogenetic analysis

## Abstract

The complete chloroplast genome of an important medicinal plant, *Convallaria majalis* Linnaeus, was sequenced for the first time. The entire circular genome is 162,218 bp in length, with 37.9% GC contents. The genome has consisted of a large single-copy region (LSC) with a length of 85,417 bp, a small single-copy region (SSC) with a length of 18,495 bp, and two inverted repeat regions (IRs) with a length of 29,153 bp each. The genome harbored 133 genes, including 87 protein coding genes, 38 tRNA genes, and eight rRNA genes. The phylogenetic tree of 24 plant species was constructed based on the maximum-likelihood method. This study will provide theoretical basis for further study on plant genetics phylogenetic research.

*Convallaria majalis* Linnaeus (1753), a perennial herb of Asparagaceae, is widely distributed in Asia, Europe and North America, growing in wet places such as shady forest or ditch according to the record of Flora Reipublicae Popularis Sinicae (FRPS). It contains many cardiac glycoside components, which provide cardiotonic and diuretic effects. Furthermore, one study in 2014 showed that odorous components derived from *C. majalis* constituted more than 20% of perfume raw material market (Dörrich et al. 2014). High content of aromatic oil in *C. majalis* possesses sweet and elegant fragrance, making it widely used in the production of soap and cosmetics. In addition to its ornamental and pharmaceutical values, *C. majalis* is known as a toxic plant. Tissue factor expression induced by saponins and various cardiac glycosides in *C. majalis* contributes to the development of a hypercoagulable state, which often led to plant poisoning among children in Finland (Lamminpää and Kinos 1996; Morimoto et al. 2021). Current studies show that steroidal glycosides derived from *C. majalis* possessed cytotoxicity to human lung adenocarcinoma cells and thus can be a potential agent for anti-lung cancer (Matsuo et al. 2017).

According to the Regulations of the People’s Republic of China on Wild Plants Protection, *C. majalis* is not in the list of national key protection of wild plants. On-site and ex-situ protection of wild plants and scientific research on wild plants are supported in article five of the regulations. With the permission of Pharmacy College in Liaoning University of traditional Chinese Medicine, *C. majalis* was identified by professor Ting-guo Kang and transplanted in the University herbal garden (E 121°53′14″, N 39°4′12″). The voucher specimen and genomic DNA were deposited at the herbarium of Liaoning University of Chinese Medicine (Liang Xu 861364054@qq.com, *C. majalis* number: 10162210515067LY). All operations are carried out in accordance with guidelines in Specification on Good Agriculture and Collection Practices for Medicinal Plants (GACP; Number: T/CCCMHPIE 2.1-2018). The extraction of total genomic DNA from fresh leaves was achieved by Magbead Plant DNA Kit (CWBIO China) and sequenced on Illumina Novaseq 6000 platform. Data were edited and assembled by NGS QC toolkit (Patel and Jain 2012) and SPAdes v3.11.0 (Bankevich et al. 2012), respectively. The protein coding sequences of chloroplast (CP) genome were compared with NR protein databases for protein-coding gene prediction and annotation.

The chloroplast genome length of *C. majalis* was 162,218 bp. The genome harbored 133 genes, including 87 protein coding genes, 38 tRNA genes, and eight rRNA genes, with a GC content of 37.9%. The genome has consisted of a large single-copy region (LSC) with a length of 85,417 bp, a small single-copy region (SSC) with a length of 18,495 bp and two inverted repeat regions (IRs) with a length of 29,153 bp each. Additionally, we find that 15 genes, including trnK-UUU, *rps*16, trnG-UCC, *atp*F, *rpo*C1, trnL-UAA, trnV-UAC, *pet*B, *pet*D, *rpl*16, *rpl*2, *ndh*B, trnI-GAU, trnA-UGC and *ndh*A, each of which contain one intron, *clp*P and *ycf*3 genes contain two introns, and *rps*12 gene has trans splicing.

Twenty-three species of plant and the outgroup *Crocus sativus* were selected for complete CP genome phylogenetic analysis according to the Bayesian information criterion (BIC). Maximum likelihood (ML) tree was constructed with the model TVM + F+R3 (Figure 1) by IQ-TREE 1.6.12 software (Nguyen et al. 2015) (bootstrap value 1000). The phylogenetic tree shows that *C. majalis* and *Convallaria keiskei* are sister groups. *Crocus sativus*, as an outgroup, is far from the other species. The species of each genus are clearly distinguished, except for *Ophiopogon bodinieri*, which is closer to the genus Liriope, since *Liriope spicata* and *Liriope muscari* were once separated from the genus Ophiopogon.

**Figure 1.**
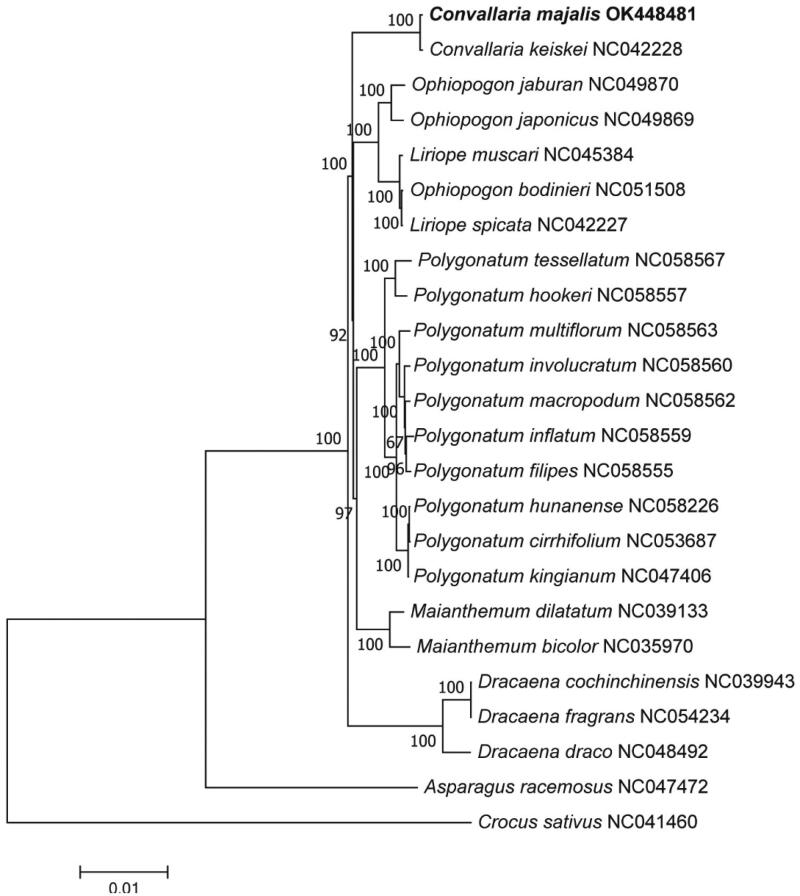
Maximum-likelihood (ML) phylogenetic tree based on the complete CP genome of *C. majalis* and 23 other species.

In conclusion, the complete CP genome of *C. majalis* was determined in this study, which provides theoretical foundation for further study on the phylogenetic relationship of Asparagaceae family.

## Data Availability

The genome sequence data that support the findings of this study are openly available in GenBank of NCBI at (https://www.ncbi.nlm.nih.gov/) under the accession NO. OK448481. The associated BioProject, SRA, and Bio-Sample numbers are PRJNA769782, SRX12547454 and SAMN22169498, respectively.
